# A model of teachers’ growth mindset, teaching enjoyment, work engagement, and teacher grit among EFL teachers

**DOI:** 10.3389/fpsyg.2023.1137357

**Published:** 2023-03-08

**Authors:** Li Liu, Jalil Fathi, Seyyed Pedram Allahveysi, Kimia Kamran

**Affiliations:** ^1^School of Marxism, Guangzhou University, Guangzhou, Guangdong, China; ^2^Department of English and Linguistics, Faculty of Language and Literature, University of Kurdistan, Sanandaj, Iran; ^3^Department of English Language and Literature, University of Ilam, Ilam, Iran

**Keywords:** teacher grit, growth mindset, teaching enjoyment, work engagement, EFL teachers

## Abstract

Because of the importance of positive emotions in second language (L2) acquisition, researchers have undertaken studies to investigate L2 learners’ emotions. Nevertheless, L2 teachers’ emotions still require more scholarly attention. Against this backdrop, we sought to test a model of teachers’ growth mindset, teaching enjoyment, work engagement, and teacher grit among English as a foreign language (EFL) teachers. To this end, 486 Chinese EFL teachers volunteered to partake in an online survey and completed the questionnaires of the four constructs in question. Confirmatory factor analysis was performed to ensure the construct validity of the used scales. Then structural equation modeling (SEM) was used to test the hypothesized model. SEM results indicated that teaching enjoyment, teacher grit, and growth mindset directly predicted EFL teachers’ work engagement. In addition, teaching enjoyment affected work engagement indirectly via the mediation of teacher grit. Likewise, teacher grit mediated the effect of growth mindset on teachers’ work engagement. Finally, the implications of these findings are discussed.

## 1. Introduction

Undoubtedly, a teacher’s career is a demanding and stressful one (Troman and Woods, 2000) and emotions play a significant role in teachers’ class achievement (Curby et al., 2013); therefore, teachers’ emotions are well-worth taking into account (Schutz, 2014; Dewaele, 2020). This has resulted in the exploration of teachers’ emotions in numerous studies (e.g., MacIntyre and Gregersen, 2012; Cuéllar and Oxford, 2018; Richards, 2020; Gregersen and MacIntyre, 2021), which have investigated various affective factors. Also, the burgeoning of the positive psychology has encouraged researchers to investigate this issue among second language (L2) researchers (Dewaele, 2005; Dewaele and MacIntyre, 2014; Dewaele et al., 2019; Shao et al., 2019; Moskowitz and Dewaele, 2020; Botes et al., 2021). As a positive affective factor, Foreign Language Teaching Enjoyment (FLTE) has recently been introduced to the literature of applied linguistics (Proietti Ergün and Dewaele, 2021) and refers to a positive feeling of joy while teaching in the class (Mierzwa, 2019). Teaching enjoyment is argued to be interrelated with teacher resilience, teacher grit, well-being, and work engagement (e.g., Proietti Ergün and Dewaele, 2021; Derakhshan et al., 2022; Xiao et al., 2022). Given the significant role of enjoyment in affecting L2 learning and its potential association with other affective constructs such as motivation, boredom, and engagement (e.g., Guo, 2021; Dewaele et al., 2022; Kruk et al., 2022), further exploration of teaching enjoyment in EFL contexts seems to be warranted.

In addition, given the difficulties and complexities of a teacher’s career, a change in one’s emotions can contribute to a change in work engagement (Ouweneel et al., 2012) which is directly correlated with the outcomes of class and student’s academic performance (Bakker and Bal, 2010). Besides, individuals are able to employ different degrees of engagement (i.e. physically, cognitively, and emotionally) in workplace due to various roles they are assigned, a construct which is referred to as work engagement (Kahn, 1990). Work engagement is concerned with involvement with full potential, abilities, competence, dedication, developmental chances, and interest in perusing goals that can foster teachers’ quality and performance (Kim et al., 2013). From this perspective, work engagement is considered as the ability to assess the desired functioning from the teachers (e.g., Schaufeli et al., 2006; Perera et al., 2018). A teacher’s work engagement has been documented as an emotionally positive one correlating with self-efficacy and reflection (Han and Wang, 2021), well-being (Rusu and Colomeischi, 2020), and students’ academic engagement (Zhang and Yang, 2021). Work engagement is also related with fulfilling positive work-related mindset (Zeng et al., 2019) and is viewed as the opposite of burnout (Bakker et al., 2005).

Furthermore, psychological thoughts and beliefs shape and reshape ones’ senses of experiences, life, attitudes, and more broadly, mindsets which significantly affect work engagement and performance in class (Nalipay et al., 2021). Mindset pertains to the beliefs regarding the mutability of one’s abilities (Dweck, 2006) which is a motivational construct (Lou and Noels, 2019). Since teachers who hold a growth mindset are more likely to get emotionally and cognitively engaged in their teaching activities and are endowed with greater overall well-being (Zeng et al., 2019), teachers’ growth mindset is hypothesized to influence their work engagement. In the meantime, conceptualized as teachers’ perseverance and passion to do their instructional activities in spite of challenges (Maiers and Sandvold, 2017), teacher grit is likely to generate positive emotions among individuals that can foster their achievements (Elahi Shirvan et al., 2021). As such, more success chances might be provided for those who are grittier than other individuals due to their consistent interests and perseverant efforts (Duckworth et al., 2009). Also, grittier individuals have a positive growth mindset that contributes to the further development of feeling of pleasure (Hill et al., 2016). Suzuki et al. (2015) claimed that grit is a powerful determiner of work engagement. Against this backdrop, it can be argued that EFL teachers’ grit can, like their growth mindset, also affect their work engagement.

Although the L2 research literature is filled with studies focusing on the impact of various affective factors on teachers’ performance (e.g., Bangun et al., 2021; Greenier et al., 2021; Li, 2021; Li et al., 2021; Zhang and Koshmanova, 2021), few sporadic studies, to our best knowledge, have investigated the joint effects of teachers’ grit, growth mindset, and their teaching enjoyment on work engagement, particularly in EFL contexts. Empirical investigations on the relationships among these factors, their nature, and the potential impact of these non-cognitive factors on teachers’ work engagement would have notable contributions to the existing body of literature related to EFL teacher emotions and characteristics. Therefore, as an attempt to shed more light on positive psychology in L2 research and also exploring antecedents of work engagement in EFL contexts, the purpose of the present researchers was to test a model of EFL teachers’ work engagement based on teaching enjoyment, teacher grit, and growth mindset.

## 2. Literature review

### 2.1. Emotions and individual differences

Learning another language is not a one-dimension processing but it is a multifaceted process in which a combination of various emotional and non-cognitive factors are also called for Oga-Baldwin et al. (2019). This has opened the gate for a plethora of studies investigating different affective factors such as self-efficacy (Young Kyo, 2022), burnout (Xing, 2022), resilience (Chaigneau et al., 2022), foreign language teaching anxiety (Aydin, 2016), self-esteem (Gaybulloyevna, 2022), boredom (Pawlak et al., 2021), psychological wellbeing (Jonas and Hall, 2022), and academic optimism (Lelieur et al., 2022). Also, key subjects of the inquiry in individual differences are beliefs and attitudes, physical and mental behaviors reflections and actions, learning strategies and styles, move and motivations, talent, mindsets, personality traits, and self-regulation (Dörnyei and Skehan, 2003). The results of the abundance of such papers led to a large number of ideas governing new rules in the field of L2 acquisition (Dewaele, 2009).

However, recent studies seem to have suppressed the old ones which used to focus only on threatening factors by shifting the idea from negative psychological factors toward positive ones (MacIntyre et al., 2019). This movement has paved the way for scholars to examine the role of individual differences and emotions under the new scope of positive psychology which has led to the emergence of numerous studies in this regard (e.g., Seligman et al., 2009; Gregersen et al., 2014; Mercer et al., 2016; Dewaele et al., 2019; Wang et al., 2021). The results of the previously mentioned studies illuminate that the process of L2 teaching can be more fruitful and L2 learning might be fostered significantly when emotions and individual differences are taken into account (Dewaele and Li, 2020).

### 2.2. Teacher mindset

The construct of mindsets or implicit/lay theories was introduced by Dweck (2006). Mindsets refer to one’s rudimentary belief systems about different human’s traits such as personality factors (Mercer and Ryan, 2010). It is widely recognized that teachers’ mindsets play a crucial role in their job achievements (Dweck, 2014). Furthermore, these mindsets tend to form and reform teachers’ beliefs of failure and success about their approach to coping with different challenges in L2 education (Cacali, 2019). Since the learning process itself is a complex one; therefore, the construct of mindset is of high significance in L2 education (Nguyen et al., 2022). Mindsets are divided into two categories of fixed and growth mindsets (Dweck et al., 1995). A fixed mindset is referred to as a belief that one’s skills, attributes, personality, and intelligence are fixed and cannot be changed whereas a growth mind is concerned with a belief that the aforementioned factors are malleable and can be enhanced (Haimovitz and Dweck, 2017). Growth mindsets refer changeable beliefs of a person of his/her own abilities when coping with setbacks (Schroder et al., 2019) which are systematically associated with endeavors, attitudes, aims, and learning strategies (Molden and Dweck, 2006). Language mindset pertains to the personal opinions about the changeability of language learning (Shirvan et al., 2021).

The studies on humans beliefs about whether they have the capacity to learn a second or foreign language have recently received much attention among L2 researchers who are interested in investigating if a person considers one’s attributes as something fixed or changeable (Lou and Noels, 2019). Mindsets are interrelated with many affective factors such as motivational factors (Lou and Noels, 2019), autonomy (Henriksen et al., 2018), wellbeing and perseverance (Zeng et al., 2019), willingness to communicate (Zarrinabadi et al., 2021), and speaking anxiety (Ozdemir and Papi, 2021). Research on learners’ mindsets seems to be well-documented and it is well acknowledged that these affective factors play a significant role in learners’ achievement; however, this topic still needs under-investigated for teachers since the literature is quite fragmented in this regard (Bostwick et al., 2020). According to Yeager and Dweck (2020), it can be argued that growth mindset’s impacts are replicable, fruitful, and theoretically well-documented for both teachers and learners. With regard to the significance of this construct in educational contexts, Zhang et al. (2017) synthesized the articles published between 1998-2017 on the relationship between learners’ academic achievement, their mindsets, and teachers’ mindsets. It was revealed that students’ mindsets play a crucial role in determining their academic achievement; however, teachers’ mindsets were found to have played a mediating role, not a determining a role. In another study, Derakhshan et al. (2022) investigated the relationships between classroom climate, growth mindset, and students’ engagement where boredom acted as a mediator. The findings indicated that learners’ growth mindset indirectly impacted students’ engagement and also, classroom climate and boredom were the significant predictors of learners’ engagement.

Additionally, it is argued that the mindset can cause shifts in one’s emotions concerning various scopes which are classified into growth mindset and fixed mindset (Yeager et al., 2022). Flexibility in teachers’ mindset is referred to as a growth mindset, whereas inflexibility in their mindset is defined as a fixed mindset. Teachers holding a growth mindset consider personality traits and personal teaching skills as a changing phenomenon (Heggart, 2015). On the other hand, those who hold a fixed mindset believe personality attributes and skills as something unmalleable, while growth mindset holders believe that smartness and intelligence can be fostered, whereas fixed mindset holders consider it as an unchanging construct (Zeng et al., 2019). Also, Frondozo et al. (2020) hypothesized that a teacher’s mindset alongside their emotions can function as a perdictor of teachers’ work engagement. The outcomes indicated that teachers with a growth teaching mindset showed a higher work engagement with teaching enjoyment as a mediating factor. In another study by Patrick and Joshi (2019), it was hypothesized that teachers’ prior ideas about the learners and learning have an impact on teachers’ mindset. It was revealed that teachers’ growth mindset is significantly correlated with students’ mindset about their own skills, personality, and attributes. The authors also found that teachers’ mindset was able to enhance or deteriorate students’ beliefs about their intelligence or skill. Also, Gutshall (2014) conducted a study on the mindset of pre-service teachers since the beginning of their practicum. The findings indicated that their mindset remained stable over their preparation program. Irie et al. (2018) also explored teachers’ mindset on their own profession. They found that teachers cared more about their own technical knowledge rather than interpersonal skills since these skills were regarded as natural.

### 2.3. Foreign language teaching enjoyment

The interest in positive psychology has paved the way for investigating positive emotions as well as the negative ones that foreign language learners and teachers might experience (Dewaele et al., 2018). This picture has changed the frame of many studies into positive psycho-affective factors (Hagenauer and Hascher, 2014). For instance, the concept of enjoyment was a reaction towards the negative factor of anxiety (Zeng, 2021). Foreign language enjoyment (FLE) can be considered as an example of positive emotions (Pekrun, 2006). It is defined as a general good feeling after overcoming an obstacle in a stressful situation (Li et al., 2018). Those who are concerned with enjoyment feel safer in various contexts and do their tasks in a controlled manner (Pekrun et al., 2007). In this vein, learning enjoyment can be defined as the joyful experience of learning through which one can develop a controlled and safe learning environment (Mierzwa, 2019). The construct of enjoyment is a multidimensional construct and includes five underlying components: *emotion, movement, cognition, eloquence, and physiology* (Hagenauer and Hascher, 2014). Among these components, emotional or the affective one refers to the pleasure feeling and the sense of joy in learning contexts, hence it fosters affective and cognitive factors in the learning environment that ultimately rises learners’ language achievement (Mierzwa, 2019).

Recently, foreign language teaching enjoyment was introduced to the literature from the studies on foreign language enjoyment by Proietti Ergün and Dewaele (2021). This construct is defined as the broad feeling of positive emotions experienced by teachers in spite of existing obstacles in the context of foreign language teaching (Noughabi et al., 2022). In this regard, Mierzwa (2019) conducted a mixed-methods study among 89 Polish teachers of English. Web questionnaires were used for data collection. It was discovered that teachers experience not only learning enjoyment, but also foreign language teaching enjoyment regardless of exterior factors such as age, residential area, or gender. In another study, Proietti Ergün and Dewaele (2021) the role of well-being and resilience as predictors of FLTE was examined among 174 Italian teachers of English as a foreign language. Their findings indicated that resilience was a strong predictor of FLTE. In another study, Xiao et al. (2022) tested a structural model of work engagement, self-efficacy, and teaching enjoyment among 315 EFL instructors. Their findings demonstrated that both predictors affected teachers’ work engagement, although teacher self-efficacy turned out to be a more powerful predictor than teaching enjoyment. Also, employing 296 English language teachers, Fathi and Naderi (2022) examined the roles of teacher resilience and FLTE in influencing teaching enjoyment. Their results indicated that FLTE was a significant predictor of teacher work engagement.

Wei et al. (2019) probed the interplay between grit, L2 performance, and L2 anxiety among 832 middle-school learners in China. The findings revealed that the learners’ grit fosters L2 performance and indirectly enhances FLE. In addition, Lee (2022) tried to shed light on the relationship between grit, classroom enjoyment, and willingness to communicate. The data analyses showed that grit and classroom enjoyment predict willingness to communicate significantly, which supports the idea that positive psychology plays a significant role in fostering the outcome of the class. Moreover, Liu and Wang (2021) discovered that grit, FLE, and L2 performance were significantly correlated with each other. Also, enjoyment was mediated by grit significantly. Moreover, Wei et al. (2019) concluded that grit impacts L2 performance greatly and enjoyment mediated between grit and L2 performance. Also, Jiang and Dewaele (2019) carried out a mixed-methods study to examine FLE and L2 anxiety of 564 EFL learners outside China. Regression analysis showed that FLE was predicted by the variables related to the teachers, while L2 anxiety was related to student-related factors.

### 2.4. Teacher grit

As previously highlighted, various studies have examined affective factors in the literature of L2 education (Dewaele, 2020). One of these psycho-affective factors that have received much attention is grit (Sudina et al., 2021). Grit, conceptualized as consistent interests and perseverant efforts for fulfilling long-term aims, can play a crucial role in learning another language (McCain, 2017). Duckworth and Quinn (2009) asserted that grit has the same level of significance as aptitude in predicting learners’ success, raising the likelihood of achieving more than the natural ability (Duckworth and Quinn, 2009). This positive social-affective trait was introduced by Duckworth and her colleagues in the University of Pennsylvania (Oxford and Khajavy, 2021). Recently, its debate has gained momentum among researchers of various disciplines (Wei et al., 2020). Applied linguistics has not been an exception and some papers (Khajavy et al., 2021; Sudina and Plonsky, 2021; Sudina et al., 2021) have investigated L2 grit among learners and teachers. This construct has been acknowledged as an important construct affecting L2 learning and teaching (Teimouri et al., 2020). Since mastering another language requires much endeavor, persistence, and enduring interest in the process of learning, grit is viewed to be a vital variable as the learners may face numerous failures (Khajavy et al., 2021). In the same vein, a teacher’s profession is full of tension and stress that leaves various impacts on teachers’ emotional state (Griffith et al., 1999). Under the pressure of teaching, teachers may either show persistence in pursuing their goals or demonstrate a lower motivation; therefore, teachers’ grit can help teachers to sustain their passion and perseverance in achieving their aims (McCain, 2017).

Concerning teachers’ grit, some studies have been conducted. For instance, Argon and Kaya (2018) explored whether the potential effects of personal factors on teachers’ grit. Their findings revealed that only gender played a pivotal role in affecting grit and the other demographic factors failed to exert significant effects on teachers’ grit. They reported low levels of teacher grit for their participants as they believed that teachers’ needs, wishes, and expectations were not met. However, a mixed-methods study on African-American male teachers conducted by Yates et al. (2015) yielded a different outcome. It was illustrated that there was a significant relationship between a teacher’s grit, age, GPA, and their life partner. In the qualitative phase, three themes were also found to be dominant, which included: (a) family, (b) circumstances, and (c) spirituality.

In a recent study, Sudina et al. (2021) tried to develop and validate a questionnaire for measuring grit among teachers. For this, 202 English language teachers were recruited as the participants. Their findings led to a valid and reliable L2-specific grit scale. Also, Azari Noughabi et al. (2022) modelled the relationship between EFL teachers’ L2 grit, engagement, and immunity. Having distributed the electronic versions of the scales among 369 EFL teachers, they found that L2 grit and engagement impacted immunity substantially. Faravani (2022) also explored teacher’s self-efficacy, grit, and continuing professional development. 204 EFL teachers were selected through convenience sampling. SEM results indicated that grit and self-efficacy acted as powerful predictors of continuing professional development. In another study, Fabelico and Afalla (2020) tested a model of teachers’ self-efficacy, grit, and burnout. Their findings indicated that grit was associated with other variables and significantly impacted teachers’ educational attainments.

Also, Nazari and Alizadeh Oghyanous (2021) investigated the relationship between job’s stress, turnover intention, wellbeing, and grit among EFL teachers. The findings revealed that these factors were significantly correlated but the associations were stronger for novice teachers. Also, the results of interviews showed that career’s stress affected emotions and perseverance which were under the impact of institutional and socioeconomic status. However, in another mixed-methods study, McCain (2017) examined the role of teachers’ grit in affecting learner’s grit and their reading achievement. Survey, observation, and interviews were used to collect the data. The findings demonstrated that there was no significant relationship between the aforementioned factors.

### 2.5. Teacher work engagement

Work engagement is related to a pleasant mindset about one’s own profession characterized by vigor, dedication, and absorption (Schaufeli and Bakker, 2004), which is associated with reflection, creativity, willingness to help colleagues, and respect towards career (Bakker, 2008). In educational contexts, teacher work engagement is defined as an affective variable indicating instructors’ voluntary devotion of physical, cognitive, and emotional resources to teaching-specific practices (Klassen et al., 2012). Scholars have introduced various models of work engagement (Choochom, 2016). For instance, Saks (2006) proposed the engagement of the employees is characterized as job and institutional involvement that might have antecedents (institutional support and critiques) and also consequences (the outcomes of work engagement). Given this, some studies (Rothmann and Hamukang’andu, 2013; Iyer, 2016; Burić and Macuka, 2018) have investigated this psycho-affective factor in educational contexts. Moreover, it is argued that more engagement on the teachers’ side contributes to the further engagement and academic achievement of the learners (Zhang and Yang, 2021).

Hultell and Gustavsson (2011) examined the factors affecting teacher burnout and engagement among Swedish beginning teachers. Their findings revealed that the demands of the job directly predicted burnout, whereas job resources were positively correlated with work engagement. Also, Hakanen et al. (2006) investigated burnout and work engagement among teachers via employing Job Demands–Resources Model in a sample of 2038 teachers. They hypothesized that there were two predictors of teacher burnout or work engagement: energetical processes (demanding tasks of a job) and motivational processes (work engagement processes). The data analysis with SEM showed that both processes affected teacher burnout or engagement; however, energetical processes predicted teacher burnout more significantly than motivational processes. In addition, motivational processes affected work engagement more significantly. Additionally, Høigaard et al. (2012) examined teacher efficacy and work engagement and their association with burnout, job satisfaction, and desire to quit among teachers with six years of experience or less. 750 questionnaires were administered to the teachers. It was found that teacher efficacy and work engagement positively predicted job satisfaction and negatively affected teacher burnout. In another study, Xiao et al. (2022) examined a structural model of the teacher self-efficacy, work engagement, and work enjoyment among EFL teachers. To this end, 315 EFL teachers completed an online survey. SEM results showed that self-efficacy and teaching enjoyment were substantial predictors of work engagement, although self-efficacy played a more significant role in affecting work engagement.

In another study, Li et al. (2015) investigated the interconnection between work engagement and value congruence. It was hypothesized that controlled and autonomous motivations play the mediating role. To this end, 767 teachers filled out the questionnaires and the data were analyzed with SEM. It was discovered that there was an indirect relationship between value congruence and work engagement and the mediating effects of the two proposed variables were also verified. In a similar study, Bakker and Bal (2010) tested the model of weekly work engagement among 54 teachers. It was hypothesized that job resources in a week are positively associated with levels of work engagement. Also, they hypothesized that momentary work engagement had a positive impact on weekly work engagement. Multilevel analyses confirmed the hypotheses that autonomy was reported as a positive predictor of work engagement. Additionally, exchanging ideas with supervisors and chances for personal growth were predictors of work engagement.

### 2.6. The hypothesized model

**FIGURE 1:**
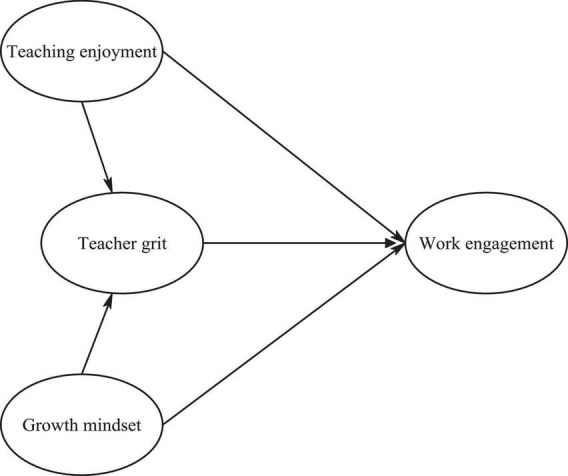
The hypothesized model of teaching enjoyment, teacher grit, growth mindset, and engagement.

As was mentioned earlier, research dealing with the association between growth mindset, teaching enjoyment, work engagement, and teacher grit still calls for further empirical inquiry. Moreover, despite that the constructs of growth mindset, teaching enjoyment, work engagement, and teacher grit have been, to greater or smaller extents, the scope of various studies carried out in the realm of foreign language education, to our best knowledge, no study has so far sought to examine the relationships between and/or among the constructs in question. Based on the theoretical and empirical considerations discussed above, a structural model, specifying the interconnections between the constructs (i.e., growth mindset, teaching enjoyment, work engagement, and teacher grit) was hypothesized and is depicted in Figure 1.

In light of the findings of Nalipay et al. (2021), it was hypothesized that teaching enjoyment directly impacts teachers’ work engagement; therefore, a path from teaching enjoyment to teachers’ work engagement was drawn. In addition, a path from teaching enjoyment to L2 teacher grit was conjectured based on the empirical study by Elahi Shirvan et al. (2021), in which enjoyment positively affected L2 grit. Also, based on the empirical findings by some studies (e.g., Singh and Chopra, 2018; Azari Noughabi et al., 2022), we hypothesized that teacher grit significantly predicts work engagement, thus, a path from grit to teaching engagement was also drawn. In addition, according to the Broaden-and-Build theory (Fredrickson, 2001) in which not only positive feelings shape emotional resources, but they also build cognitive ones which can serve as pacifiers for long-term negative emotions, and also consistent with the findings of Hochanadel and Finamore (2015), we hypothesized that growth mindset can impact teacher grit. Moreover, in light of the nature of the construct and some previous studies (e.g., O’Neal et al., 2018; Ma et al., 2020; Lan et al., 2021; Lee and Ha, 2022), we hypothesized that grit acts as the mediator for the impact of teaching enjoyment on teaching engagement. Likewise, due to the characteristics of the constructs and based on the findings of some studies (e.g., Özhan, 2021; Kim et al., 2022), it was hypothesized that grit mediates between the impact of growth mindset on work engagement of EFL teachers. Concerning the hypothesized model, the following hypotheses were proposed:

Hypothesis 1: Teaching enjoyment significantly predicts work engagement.

Hypothesis 2: L2 teacher grit. positively affects teachers’ work engagement.

Hypothesis 3: Growth mindset positively influences work engagement.

Hypothesis 4: Grit mediates the effect of teaching enjoyment on teachers’ work engagement.

Hypothesis 5: Grit mediates the effect of growth mindset on teachers’ work engagement.

## 3. Materials and methods

### 3.1. Participants

In this study, a total number of 486 Chinese EFL teachers took part as the participants. Among the participants, 189 were male and 297 were female English instructors who were engaged in teaching English at schools, universities, and language academies in Chinese provinces. Given availability, convenient access, and willingness criteria, the participants were selected based on convenience sampling. They were selected based on convenience sampling procedure. Their age range fell between 21 and 44 years (*M* = 25.03, *SD* = 6.74) and their teaching experience varied from 2 to 21 years of teaching experience (*M* = 7.21, *SD* = 5.37).

### 3.2. Measures

#### 3.2.1. Teaching enjoyment

In order to measure teaching enjoyment of the participants, the Foreign Language Teaching Enjoyment Scale (FLTES) validated by Proietti Ergün and Dewaele (2021) was used in this study. FLTES includes nine statements which assess three underlying components: 1) Personal Enjoyment (PE), 2) Student Appreciation (SA), and 3) Social Enjoyment (SE). The items are measured on a 5-point Likert scale varying from 1 (strongly disagree) to 5 (strongly agree). A sample item is “The students are stimulating.”

#### 3.2.2. Teacher grit

The grit level of the EFL instructors was assessed with the scale validated by Sudina et al. (2021). This self-report scale contains 14 statements which measure two dimensions of L2 teaching grit including consistency of interest (CI) and perseverance of effort (PE). The items are assessed on a 5-point scale from 1 (*not like me at all*) to 5 (*very much like me*). A sample item is “As an ESL/EFL teacher, I am diligent.”

#### 3.2.3. Work engagement

Work engagement of EFL teachers participating in this study was gauged with the *Engaged Teacher Scale* (ETS) validated by Klassen et al. (2013). The questionnaire includes 16 statements, rated on a 7- point Likert scale (e.g. 1 = never, 7 = always), which measure four dimensions: *cognitive engagement*, *emotional engagement*, *students’ social engagement*: students, and *colleagues’ social engagement*. The psychometric properties of this scale have been approved by the previous researchers (e.g., Topchyan and Woehler, 2021). A sample item is “I feel happy when I am working intensely.”

#### 3.2.4. Growth mindset

We measured the growth mindset of EFL teachers by adopting the six items originally used in Dweck’s (2014) mindset questionnaire. The items of this self-report scale are measured on a 6-point Likert scale varying from 1 (strongly disagree) to 6 (strongly agree). A sample item is “You can always substantially change how intelligent you are.”

### 3.3. Data collection

As the first step to collect the data for this non-experimental study, an electronic survey which included a section for demographic information as well as another section for the four scales was constructed using WeChat application. Creating the electronic version of the questionnaires helped the researchers to collect data from various regions in China. The link of the questionnaires was shared on a large-scale with various teachers in online space. Also, some colleagues and friends cooperated with the first researcher in data collection. As the participants were English teachers and possessed an adequate command of English proficiency, the English versions of the scales were used and there was no need to translate them into Chinese. The necessary explanation on how to complete the items was included in the beginning of the electronic survey. Informed consents were obtained from all participants who were also ensured about the confidentiality of their data. The participation was also announced to be voluntary and the participants could give up at any stage that they wished. Overall, the data collection started in March 2022 and took about 9 weeks to collect all the data.

### 3.4. Analytic procedure

SPSS 22 and AMOS 23 were used to analyze the collected data in this study. Then SEM was employed to test the hypothesized model in this study. Prior to performing SEM, confirmatory factor analysis (CFA) was used to test the measurement models in order to verify the construct validity of the used scales. Regarding the evaluation of the model fit, a number of goodness-of-fit indices were used: Chi-square divided by degree of freedom (χ^2^/df), Comparative Fit Index (CFI), Tucker–Lewis Index (TLI), and Root Mean Square Error of Approximation (RMSEA). A model is regarded to have acceptable fit in case χ^2^/df < 3, CFI and TLI ≥ 0.90, and RMSEA ≤ 0.08 (Kline, 2011).

## 4. Results

### 4.1. Pre-processing of the data

As the initial screening, the collected data were checked for missing values, outliers, and non-normal data. Concerning the missing data, we employed Expectation-Maximization algorithm as a data imputation technique (Kline, 2011). Univariate outliers were determined by converting all the scores to standard scores for each construct. Moreover, we used Mahalanobis D^2^ to determine multivariate outliers. Skewness and kurtosis indices were used to examine the data normality and values falling out of ±2.0 were treated as non-normal data (Kunnan, 1998). The outliers and non-normal values were discarded prior to running CFA and SEM. Table 1 depicts the number of valid cases for each construct.

**TABLE 1:** Number of cases for each measure.

	No of original cases	No of outliers	No of missing cases	No of valid cases
Self-efficacy	486	3	2	481
School climate	486	4	3	479
Wellbeing	486	3	3	480
FLTE	486	2	2	482

### 4.2. Validity and reliability of the scales

To verify the validity of the scales used in this research, the measurement models were tested via conducting CFA. Table 2 indicates the goodness of fit indices for each construct.

**TABLE 2:** Measurement model of the latent variables.

	χ^2^	df	χ^2^/df	CFI	TLI	RMSEA	Cronbach’s α
Teaching enjoyment	184.32	85	2.16	0.92	0.93	0.06	0.83
Teacher grit	88.71	43	2.06	0.91	0.92	0.07	0.79
Growth mindset	97.33	50	1.94	0.96	0.95	0.05	0.91
Work engagement	412.25	210	1.96	0.95	0.95	0.05	0.81

As some models failed to demonstrate adequate fit to the data, some modifications were made to the models, in which included the omission of two items of work engagement, two teacher grit items, and one item of teaching enjoyment scales as these items’ factor loadings were low. The revised model indicated good fit to the data (see Table 3).

**TABLE 3:** Fit indices for the initial and revised models.

	χ^2^	Df	χ^2^/df	CFI	TLI	RMSEA
Initial model	402.31	196	2.05	0.92	0.91	0.06
Revised model	352.14	182	1.93	0.95	0.95	0.04

Concerning the internal consistency of the questionnaires, the computed coefficient alphas were all higher than 0.70, approving the acceptability of their reliability (Hair et al., 1998) (see Table 2). Then, descriptive statistics and correlations among the constructs were calculated (Table 4).

**TABLE 4:** Descriptive statistics and correlations.

	*M* (SD)	1	2	3	4
(1) Teaching enjoyment	3.71 (.92)	1.00			
(2) Teacher grit	3.98 (.89)	0.38**	1.00		
(3) Growth mindset	3.91 (.94)	0.25*	0.33**	1.00	
(4) Work engagement	4.12 (1.02)	0.51**	0.36**	0.32**	1.00

*p < 0.05. **p < 0.01.

### 4.3. Model testing

**FIGURE 2:**
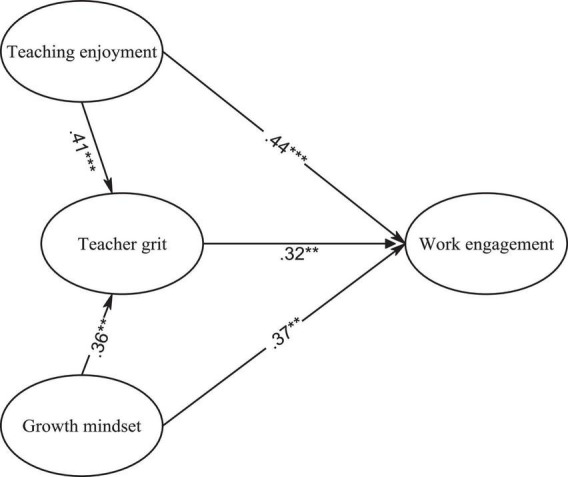
The final model of EFL teachers’ work engagement. **p < 0.01. ***p < 0.001.

Employing the maximum likelihood technique and variance-covariance matrices as input, we tested the hypothesized model via AMOS program. The calculated fit indices demonstrated a good fit of the model to the data, thereby confirming all the hypotheses in the final model (see Figure 2). Also, effect size (ES) (Cohen’s *f*^2^) was calculated to gain a better understanding of the obtained results.

As illustrated in Figure 2, teaching enjoyment, grit, and growth mindset were the directly predicted work engagement of EFL teachers. Among these constructs, teaching enjoyment was the strongest direct predictor of work engagement (β = 0.44, *R*^2^ = 0.19, *f*^2^ = 0.23, large effect size). Growth mindset (β = 0.37, *R*^2^ = 0.13, *f*^2^ = 0.15, medium effect size) and teacher grit (β = 0.32, *R*^2^ = 0.10, *f*^2^ = 0.11, medium effect size) also directly predicted teachers’ work engagement. In addition, teaching enjoyment influenced work engagement indirectly through the mediation of teacher grit (β = 0.41 × 0.32 = 0.13, *R*^2^ = 0.001, *f*^2^ = 0.001, small effect size). Likewise, growth mindset impacted work engagement indirectly through teacher grit (β = 0.36 × 0.32 = 0.11, *R*^2^ = 0.01, *f*^2^ = 0.01, small effect size). Also, it was revealed that teaching enjoyment (β = 0.41, *R*^2^ = 0.16, *f*^2^ = 0.20, large effect size) and growth mindset (β = 0.36, *R*^2^ = 0.12, *f*^2^ = 0.14, medium effect size) had significant effects on teacher grit.

## 5. Discussion

The present study aimed at testing a structural model specifying the associations between teaching enjoyment, growth mindset, teacher grit, and work engagement among Chinese EFL teachers. First, the construct validity of the used scales was confirmed through performing CFA. Then, SEM analyses used to test the hypothesized model yielded several significant findings.

First, teaching enjoyment was found to be a significant predictor of a teachers’ work engagement, supporting Hypothesis 1. This finding is in line with the outcomes reported by previous researchers (e.g., Ouweneel et al., 2012; Datu et al., 2022; Elkheloufi and Yean, 2022; Xiao et al., 2022) who demonstrated that positive emotions including feeling of joy and pleasure play a crucial role in affecting work engagement. The contribution of the teaching enjoyment to teachers’ engagement can be interpreted with regard to the fact that teachers who do not get bored, feel pleasure in the class, and enjoy the good atmosphere of the class are more likely to be engaged in their teaching activities (Burić and Macuka, 2018). Also, our findings partially support the results of Hakanen et al. (2006), Yapar and Ince (2014), and Li et al. (2021) who found that teaching enjoyment processes negatively affected teacher burn out, which is the opposite counterpart of work engagement (Zeng, 2021).

Second, SEM analysis supported Hypothesis 2 by indicating that L2 grit positively affected EFL teachers’ work engagement. This finding mirrors the results reported in the literature (e.g., Suzuki et al., 2015; Nazari and Alizadeh Oghyanous, 2021; Azari Noughabi et al., 2022), which have considered L2 grit as a negative predictor of teachers’ job stress and a positive correlate of their engagement and wellbeing. Furthermore, the positive association between grit and work engagement can be justified by the relationship between the elements of grit, consistency of desire and perseverance, and elements of work engagement, attention and enthrallment (Singh and Chopra, 2016). In addition, this finding, in part, resonates with Fabelico and Afalla (2020) who reported that teachers’ grit positively impacts their educational achievements. Also, the finding of this study resonates with those of Hodge et al. (2018), Wei et al. (2019), Liu and Wang (2021) who reported that grit significantly predicts engagement. In other words, we argue that EFL teachers who show further consistent interest and perseverant efforts in their teaching activities are more likely to get more emotionally, cognitively, and physically involved in their teaching activities.

Third, it was discovered that growth mindset significantly predicted teachers’ work engagement; therefore, Hypothesis 3 was supported. The positive effect of growth mindset on work engagement is in accordance with some studies reported in the literature (Zeng et al., 2016; Caniëls et al., 2018; Patrick and Joshi, 2019; Frondozo et al., 2020; Nalipay et al., 2021; Elkheloufi and Yean, 2022) in which growth mindset possessors show higher work engagement than fixed mindset holders. Therefore, such teachers are more optimistic about enhancing their performance level, leading to their heightened engagement in their teaching practices. Also, the finding of the present study partially accords with the study by Fan (2022) who concluded that the growth mindset plays a mediating role between a teacher’s positive psycho-affective variables and work engagement, the findings which was also corroborated by Elkheloufi and Yean (2022). Furthermore, our findings partially support those of Zhao et al. (2021) in which growth mindset indirectly and positively influences work engagement.

Fourth, SEM results indicated that teaching enjoyment affected work engagement through teacher grit (enjoyment → grit → engagement), supporting hypothesis 4. This finding is partially in line with that of Derakhshan et al. (2022) who reported associations among teacher grit, well-being, and foreign language teaching enjoyment. This finding can also be justified in light of Broaden-and-Build theory (Fredrickson, 2001) which posits that positive emotions can broaden individuals’ personal resources and foster innovative, exploratory thoughts and actions. From this perspective, we argue that teaching enjoyment as a pleasant emotion broadens teachers’ behavioral repertoire and builds their skills and psychological resources, leading their increased consistency of interests and their perseverance of efforts in their teaching practices. This in turn leads to teachers’ greater engagement in their instructional activities.

Finally, SEM results indicated that teacher grit mediated the effect of growth mindset on teachers’ work engagement (growth mindset → grit → engagement), supporting hypothesis 5. This finding is consistent with that of Zeng et al. (2019) who found teachers’ growth mindset affects their work engagement with the mediating effects of well-being and grit. Likewise, Frondozo et al. (2020) also reported that teacher’s growth mindset affects their enjoyment and engagement. Likewise, such findings have been verified by Nalipay et al. (2021) who found significant interconnections among teachers’ growth mindset, motivation, and engagement. We argue that teachers who believe in the malleability of their competencies devote further efforts and interest (i.e., grit) to enhancing their teaching quality, which in turn leads to their being more emotionally, cognitively, and agentically attached to their teaching practices.

## 6. Conclusion and implications

In the present study, we aimed to test a structural model of teachers’ growth mindset, foreign language teaching enjoyment, teacher engagement, and teaching grit. To our best knowledge, this is the first study to examine the interplay among these latent variables at the same time. For this purpose, first, the construct validity of the constructs was confirmed via performing CFA. Then, SEM was employed to test the hypothesized model specifying the relationships between these variables. Data analysis revealed that foreign language teaching enjoyment, teaching grit, and growth mindset affected a teacher’s work engagement directly and significantly. Also, teacher grit mediated the effects of teaching enjoyment and growth mindset on the work engagement.

Due to the important impact of a teacher’s work engagement on the class achievement (Perera et al., 2018; Slišković et al., 2019; Zeng et al., 2019), this construct is worth taking into consideration. Therefore, the impact of various psycho-affective factors affecting this construct needs to be investigated and included in teacher education programs to foster teachers’ work engagement and foster this variable to train more engaged teachers and ultimately, enhance the quality of the instruction. Moreover, according to our obtained findings, teaching enjoyment was revealed to directly and positively affect teachers’ work engagement. In other words, if teachers hold positive emotions regarding their teaching activities, they will be more engaged in their teaching activities. As a result, it is recommended that teacher educators take initiatives in order to enhance positive emotions of pre-service teachers in their programs so as to raise teachers’ work engagement. More in line with our findings, EFL teacher trainers should focus on the potential avenues to foster teachers’ teaching enjoyment, grit, and growth mindset so that they can enhance their work engagement in L2 instruction which is intrinsically a demanding enterprise. Also, teaching grit was discovered as another significant factor affecting teachers’ work engagement and a mediator affecting the constructs in our model. Given these findings, teacher educators and policy makers, especially in Chinese EFL context, should also think about the practical steps in order to enhance teachers’ grit in accomplishing their instructional goals in EFL contexts. Moreover, policy makers, stake-holders, and administrators should take teachers’ grit and growth mindset into account in their recruitment criteria as these two constructs were found to affect EFL teachers’ work engagement.

Concerning the study limitations some points should be taken into account. The researchers only used quantitative research methods to gather the data. However, using self-report measures are less likely to give in-depth understanding of the nature of the associations among the latent constructs such as enjoyment, grit, growth mindset, and work engagement. As such, future researchers are encouraged to replicate similar studies by employing qualitative methods to triangulate their quantitative findings and shed more light on the interconnections among these constructs. The sample recruited in this study was not a big sample, thereby limiting the generalizability of the findings. Therefore, future researchers are recommended to recruit larger samples from other EFL contexts to increase the generalizability of the findings.

## Data availability statement

The original contributions presented in this study are included in the article/supplementary material, further inquiries can be directed to the corresponding author.

## Ethics statement

The studies involving human participants were reviewed and approved by University of Kurdistan, Iran. The patients/participants provided their written informed consent to participate in this study.

## Author contributions

LL gathered the data in China and proposed the variables, wrote an initial draft of the manuscript. JF proposed the design, analyzed the data, and supervised the coordination among authors. SA wrote the Introduction and Literature review sections and edited the other sections. KK wrote the Discussion section, did the data pruning, and edited the other sections. All authors contributed to the article and approved the submitted version.
